# Relationship and distribution of *Salmonella enterica* serovar I 4,[5],12:i:- strain sequences in the NCBI Pathogen Detection database

**DOI:** 10.1186/s12864-022-08458-z

**Published:** 2022-04-06

**Authors:** Julian M. Trachsel, Bradley L. Bearson, Brian W. Brunelle, Shawn M. D. Bearson

**Affiliations:** 1 Food Safety and Enteric Pathogens Research Unit, National Animal Disease Center, Agricultural Research Service, United States Department of Agriculture, Ames, Iowa USA; 2 Oak Ridge Institute for Science and Education, Agricultural Research Service Participation Program, Oak Ridge, TN USA; 3 Agroecosystems Management Research Unit, National Laboratory for Agriculture and the Environment, Agricultural Research Service, United States Department of Agriculture, Ames, Iowa USA; 4 Present Address: Present address: Arbor Biosciences, Ann Arbor, MI USA

**Keywords:** *Salmonella enterica* serovar I 4,[5],12:i:-, NCBI Pathogen Detection, *Salmonella* Genomic Island 4, Antimicrobial resistant, Multidrug-resistant, Metal tolerance, Integrative and conjugative element, *sopE*

## Abstract

**Background:**

Of the > 2600 *Salmonella* serovars, *Salmonella enterica* serovar I 4,[5],12:i:- (serovar I 4,[5],12:i:-) has emerged as one of the most common causes of human salmonellosis and the most frequent multidrug-resistant (MDR; resistance to ≥3 antimicrobial classes) nontyphoidal *Salmonella* serovar in the U.S. Serovar I 4,[5],12:i:- isolates have been described globally with resistance to ampicillin, streptomycin, sulfisoxazole, and tetracycline (R-type ASSuT) and an integrative and conjugative element with multi-metal tolerance named *Salmonella* Genomic Island 4 (SGI-4).

**Results:**

We analyzed 13,612 serovar I 4,[5],12:i:- strain sequences available in the NCBI Pathogen Detection database to determine global distribution, animal sources, presence of SGI-4, occurrence of R-type ASSuT, frequency of antimicrobial resistance (AMR), and potential transmission clusters. Genome sequences for serovar I 4,[5],12:i:- strains represented 30 countries from 5 continents (North America, Europe, Asia, Oceania, and South America), but sequences from the United States (59%) and the United Kingdom (28%) were dominant. The metal tolerance island SGI-4 and the R-type ASSuT were present in 71 and 55% of serovar I 4,[5],12:i:- strain sequences, respectively. Sixty-five percent of strain sequences were MDR which correlates to serovar I 4,[5],12:i:- being the most frequent MDR serovar. The distribution of serovar I 4,[5],12:i:- strain sequences in the NCBI Pathogen Detection database suggests that swine-associated strain sequences were the most frequent food-animal source and were significantly more likely to contain the metal tolerance island SGI-4 and genes for MDR compared to all other animal-associated isolate sequences.

**Conclusions:**

Our study illustrates how analysis of genomic sequences from the NCBI Pathogen Detection database can be utilized to identify the prevalence of genetic features such as antimicrobial resistance, metal tolerance, and virulence genes that may be responsible for the successful emergence of bacterial foodborne pathogens.

**Supplementary Information:**

The online version contains supplementary material available at 10.1186/s12864-022-08458-z.

## Background

*Salmonella enterica* serovar I 4,[5],12:i:- (serovar I 4,[5],12:i:-) is a monophasic variant of serovar Typhimurium whose prevalence has increased in the United States and globally over the last 2 decades. Analysis of data from the National Antimicrobial Resistance Monitoring System (NARMS) in the U.S. indicates that the prevalence of serovar I 4,[5],12:i:- as a proportion of all nontyphoidal *Salmonella* (NTS) serovars increased 3.1-fold between 2003 (2%) and 2015 (6.3%) [1, 2]. Of the > 2600 serovars of NTS, serovar I 4,[5],12:i:- was the 4th most common serovar in 2015, an increase from 10th in 2003. Furthermore, multidrug-resistance of serovar I 4,[5],12:i:- isolates in the NARMS data has increased 8-fold between 2004 (8.3%) and 2015 (67.8%), resulting in serovar I 4,[5],12:i:- being the most frequent multidrug-resistant (MDR; resistance to ≥3 antimicrobial classes) nontyphoidal *Salmonella* serovar in the U.S. for years 2013, and 2015–2018 (34.5% of all nontyphoidal serovars in 2015) [3, 4]. For 2016–2018, 4.5% of NTS were serovar I 4,[5],12:i:- strains, 26% of MDR NTS were MDR serovar I 4,[5],12:i:- isolates, and 54% of serovar I 4,[5],12:i:- strains were MDR.

In 2015, a multistate (WA, OR, ID, CA, AK) outbreak of MDR serovar I 4,[5],12:i:- associated with consumption of contaminated pork resulted in 188 human infections with 30 hospitalizations [5]. The MDR serovar I 4,[5],12:i:- isolates linked to the foodborne outbreak encoded the *bla*_TEM − 1_, *strAB*, *sul2*, and *tet*(B) genes for resistance to ampicillin, streptomycin, sulfisoxazole, and tetracycline (R-type ASSuT). The outbreak resulted in a recall of 523,380 lbs. of pork.

Recently, we described the genome sequence (NCBI accession CP040686) of serovar I 4,[5],12:i:- strain USDA15WA-1 (FSIS1503788), which was isolated from swine cecal contents collected by the USDA, Food Safety and Inspection Service from the slaughter plant associated with the 2015 U.S. pork outbreak investigation [6]. Strain USDA15WA-1 does not contain any plasmids including the virulence plasmid (pSLT) that is often present in isolates of serovar Typhimurium. An MDR module [7] conferring the R-type ASSuT is inserted in the *fljB* region of USDA15WA-1 and deletes an ~15 kb genomic segment including *fljB* compared to serovar Typhimurium; this deletion results in the monophasic phenotype for strain USDA15WA-1. In addition to antimicrobial resistance genes, the MDR module contains multiple genes encoding tolerance to mercury (Hg). An ~80 kb genetic element termed *Salmonella* Genomic Island 4 (SGI-4) [8] is inserted in the *pheR* tRNA of strain USDA15WA-1 and encodes additional heavy metal tolerance genes (HMTGs) for copper (Cu), arsenic (As), and silver (Ag); the presence of SGI-4 in serovar I 4,[5],12:i:- strains from the U.K., Japan, and the U.S. has been shown to enhance *Salmonella* tolerance to copper, arsenic, and antimony [9–11]. Furthermore, exposure to 2 mM copper reduced bacterial motility significantly less in serovar I 4,[5],12:i:- strains containing HMTGs compared with isolates lacking the metal tolerance loci [12]. Investigations [9–11] have also demonstrated that SGI-4 is an integrative and conjugative element (ICE) that can be transferred to other bacterial strains by conjugation and confer increased metal tolerance in recipient isolates. DNA sequence analysis indicated that the MDR module of strain USDA15WA-1 was similar to serovar I 4,[5],12:i:- strain 07–2006 [7], and both the MDR module and the SGI-4 ICE were similar to serovar I 4,[5],12:i:- isolates SO4698–09 [8], TW-Stm6 [13] and L-4234 [14] which were isolated in Germany, the United Kingdom, Australia, and Japan, respectively. Our data on the genome sequence of serovar I 4,[5],12:i:- strain USDA15WA-1 suggest that this 2015 pork outbreak-associated strain is related to strains that are globally distributed including Europe, Australia, and Asia. In the current study, we compared the genome sequence of strain USDA15WA-1 to determine its relatedness to other *Salmonella* serovar I 4,[5],12:i:- strains whose genome sequences were deposited in the NCBI Pathogen Detection database. Furthermore, we examined the distribution of the *Salmonella* serovar I 4,[5],12:i:- strain sequences as well as the prevalence of the MDR module, SGI-4, and antimicrobial resistance profiles in these genomes. Finally, we identified clusters of closely related isolates and used this information to investigate potential sources of this important foodborne pathogen.

## Results and discussion

### Distribution and phylogenetic relatedness of *Salmonella* serovar I 4,[5],12:i:- strains in NCBI Pathogen Detection database

**Fig. 1:**
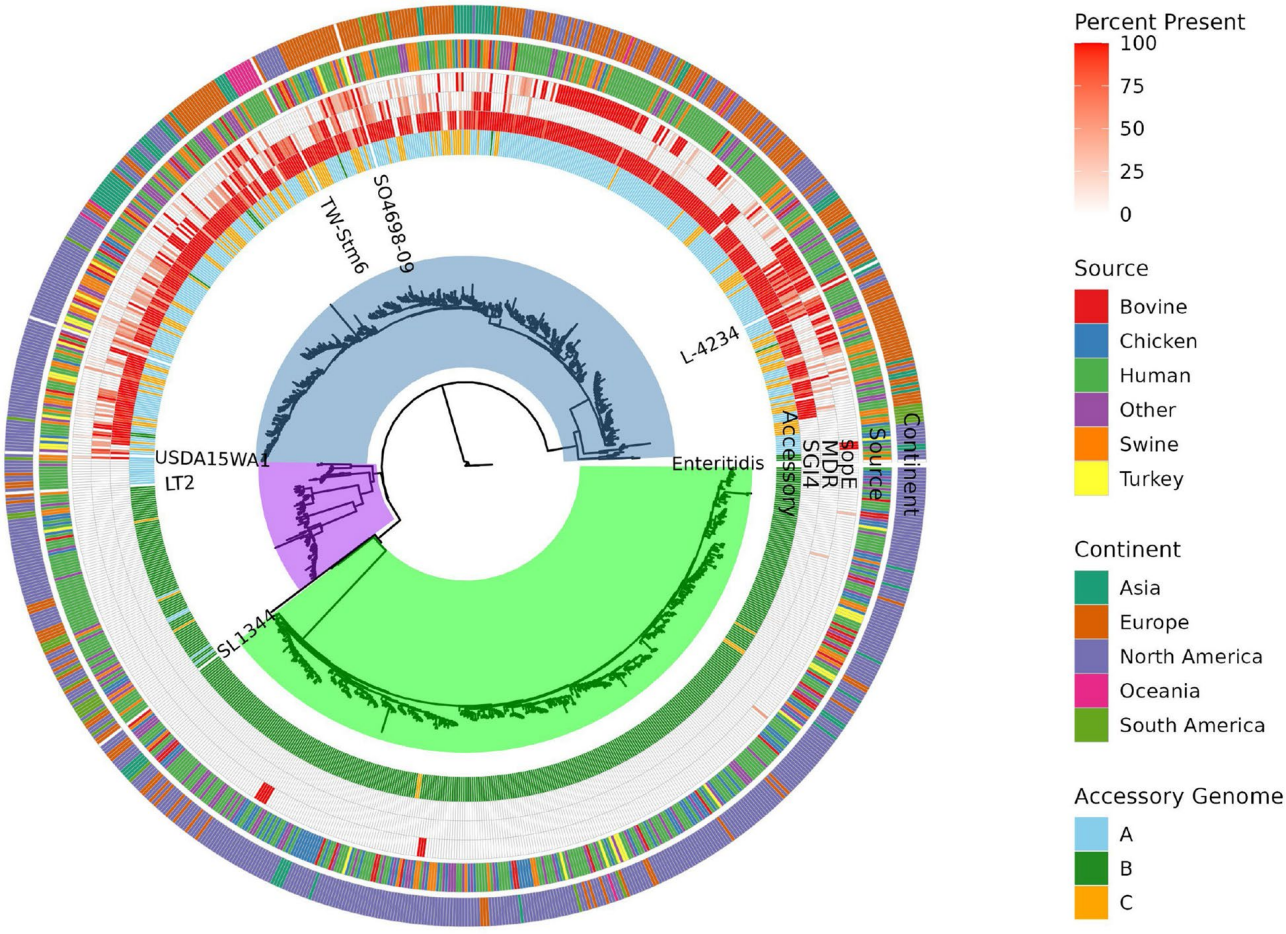
Phylogenetic tree depicting relationship between 912 groups of genomes representing 13,612 serovar I 4,[5],12:i:- strain sequences from the NCBI Pathogens database compared to genome sequences of *Salmonella* serovar I 4,[5],12:i:- (TW-STM6, USDA15WA-1, L-4234, and SO4698–09), serovar Typhimurium (LT2 and SL1344), and serovar Enteritidis (92–0392) reference strains. The number of cluster representatives in clades 1, 2, and 3 was 447, 96, and 364, respectively. Successive rings on the tree from outside to inside were propagated from metadata in Supplemental Table 1 and indicate continent of origin (continent), source (source), percent presence of specific genetic elements (*sopE* gene (targ_presence_SO4698_sopE_mTmV), MDR module (targ_presence_MDR), and SGI-4 (targ_presence_SGI4)), and accessory genome cluster. The center area of the figure indicates strain sequences associated with clades 1 (blue), 2 (purple), and 3 (green)

**Fig. 2:**
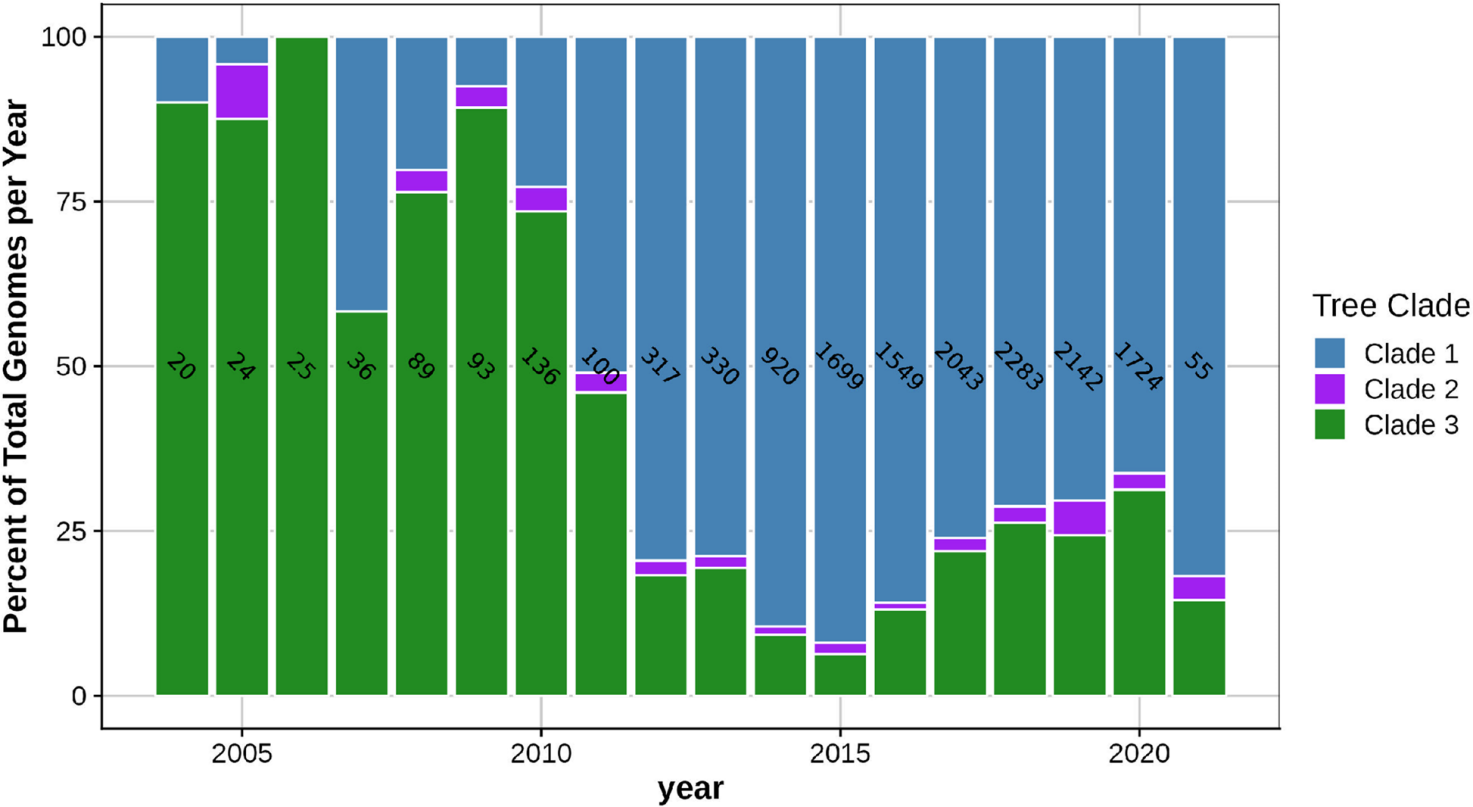
The percent of serovar I 4,[5],12:i:- strain sequences in clades 1, 2, and 3 each year. The number of strain sequences for each isolation year is indicated within a bar

A total of 13,612 assemblies of serovar I 4,[5],12:i:- genomes meeting our criteria (< 200 contigs and total assembly length < 5.7mb) were available in the NCBI Pathogen Detection database, and these strain sequences represented 30 countries from 5 continents (North America, Europe, Asia, Oceania, and South America), but were predominately from the United States and the United Kingdom. Multiple *Salmonella* strains previously described in the literature were used as reference genomes for genomic comparisons including serovar I 4,[5],12:i:- (TW-STM6, L-4234, SO4698–09, and USDA15WA-1), serovar Typhimurium (LT2 and SL1344), and serovar Enteritidis (92–0392) [6, 8, 13, 14]. The metadata in Supplemental Table 1 indicates the closest reference strain for each of the 13,612 serovar I 4, [5],12:i:- sequences extracted from the NCBI Pathogen Detection database, and a phylogenetic tree demonstrating the relationship between representative strain sequences is shown in Fig. 1. The maximum likelihood phylogenetic tree based on a core genome alignment separated serovar I 4,[5],12:i:- sequences into 3 clades that we termed clade 1, clade 2, and clade 3. Isolate sequences related to the serovar I 4,[5],12:i:- reference strains TW-STM6 (3722), USDA15WA-1 (3423), L-4234 (1725), and SO4698–09 (1360) segregated to clade 1 (Supplemental Table 2), which contained by far the most strain sequences (10,230; 75%) that were predominately isolated over the last decade (Fig. 2). All sequences in clades 2 and 3 were related to serovar Typhimurium strains LT2 (343; 2.5%) and SL1344 (3039, 22%), respectively. Serovar Enteritidis strain 92–0392 formed the root of the phylogenetic tree and no serovar I 4,[5],12:i:- isolate sequences were included in the root. The phylogenetic relatedness patterns of serovar I 4,[5],12:i:- isolates in this collection suggest that while a majority of known strains belong to a single closely related clade, at least two other lineages also exist and that isolates serotyped as “I 4,[5],12:i:-” do not necessarily represent a genetically homogeneous population.

Isolate sequences from the NCBI Pathogen Detection database related to the individual reference strains tended to have an isolation source associated with a predominant continent/country as shown on the outer ring of the phylogenetic tree (Fig. 1). For example, clade 1 sequences closely related to serovar I 4, [5],12:i:- reference strain USDA15WA-1, originally isolated in the U.S. from swine cecal contents, were almost exclusively (99.9%) isolated from countries in North America and were predominately (95%) from the U.S. (Supplemental Table 2). Similarly, clade 1 strain sequences closely related to serovar I 4, [5],12:i:- reference strain SO4698–09, originally isolated from a bovine sample in the U.K., were almost exclusively (99.3%) isolated in European countries and 92% of these were from the U.K. Clade 1 strain sequences closely related to serovar I 4,[5],12:i:- reference strain L-4234, originally isolated from a swine sample in Japan, were frequently isolated from countries in Europe (87.5%), with the majority (86%) coming from the U.K. Clade 3 strain sequences closely related to serovar Typhimurium reference strain SL1344 were predominately isolated from countries in North America (96%) and 85% of these were from the U.S. Although relatively small in comparison to clades 1 and 3, clade 2 strain sequences most closely related to serovar Typhimurium reference strain LT2 predominately originated from countries in North America [77% (U.S. 91%)] and Europe [16% (U.K. 96%)]. Clade 1 strain sequences closely related to serovar I 4,[5],12:i:- reference strain TW-STM6, originally isolated from swine feces in Australia, included the largest number of strains in the dataset and also had a greater distribution across multiple continents compared to sequences related to other reference strains. However, in this collection, North America [50% (U.S., 97.6%)] and Europe [38% (U.K., 85.8%)] remained the dominant isolation sources for strain sequences related to strain TW-STM6. The patterns described here show that various lineages of strains typed as serovar I 4,[5],12:i:- have been isolated from diverse geographic locations, and that some closely related strains appear to have distributions that span multiple continents.

### Serovar I 4,[5],12:i:- sequence types

The 13,612 serovar I 4,[5],12:i:- isolates were composed of 25 multilocus sequence types (ST); sequence types ST34 (9589), ST19 (3355) and ST2379 (480) were the most predominant and were represented by 99% of sequences. Serovar I 4,[5],12:i:- isolates SO4698–09 [8] and L-4234 [14] were previously shown to be ST34 and the NCBI metadata classified USDA15WA-1 as ST2379. The majority of strain sequences most closely related to SO4698–09 (99%), L-4234 (97%), and TW-STM6 (95%) were classified as ST34. Strain sequences related to USDA15WA-1 were most frequently ST34 (88%), but 10% were ST2379. The majority of strain sequences most closely related to SL1344 (99%) and LT2 (97%) were ST19. Other serovar I 4,[5],12:i:- sequence types included ST2078 (1), ST2079 (1), ST2956 (15), ST3119 (1), ST3168 (2), ST3224 (28), ST3228 (4), ST3343 (1), ST3478 (6), ST3545 (3), ST3562 (1), ST3591 (1), ST3777 (1), ST3805 (18), ST4028 (6), ST4067 (2), ST4081 (1), ST4281 (1), ST4431 (13), ST4452 (1), ST4650 (1), ST4734 (1), and unclassified (79).

### Serovar I 4,[5],12:i:- host and clade association

**Fig. 3:**
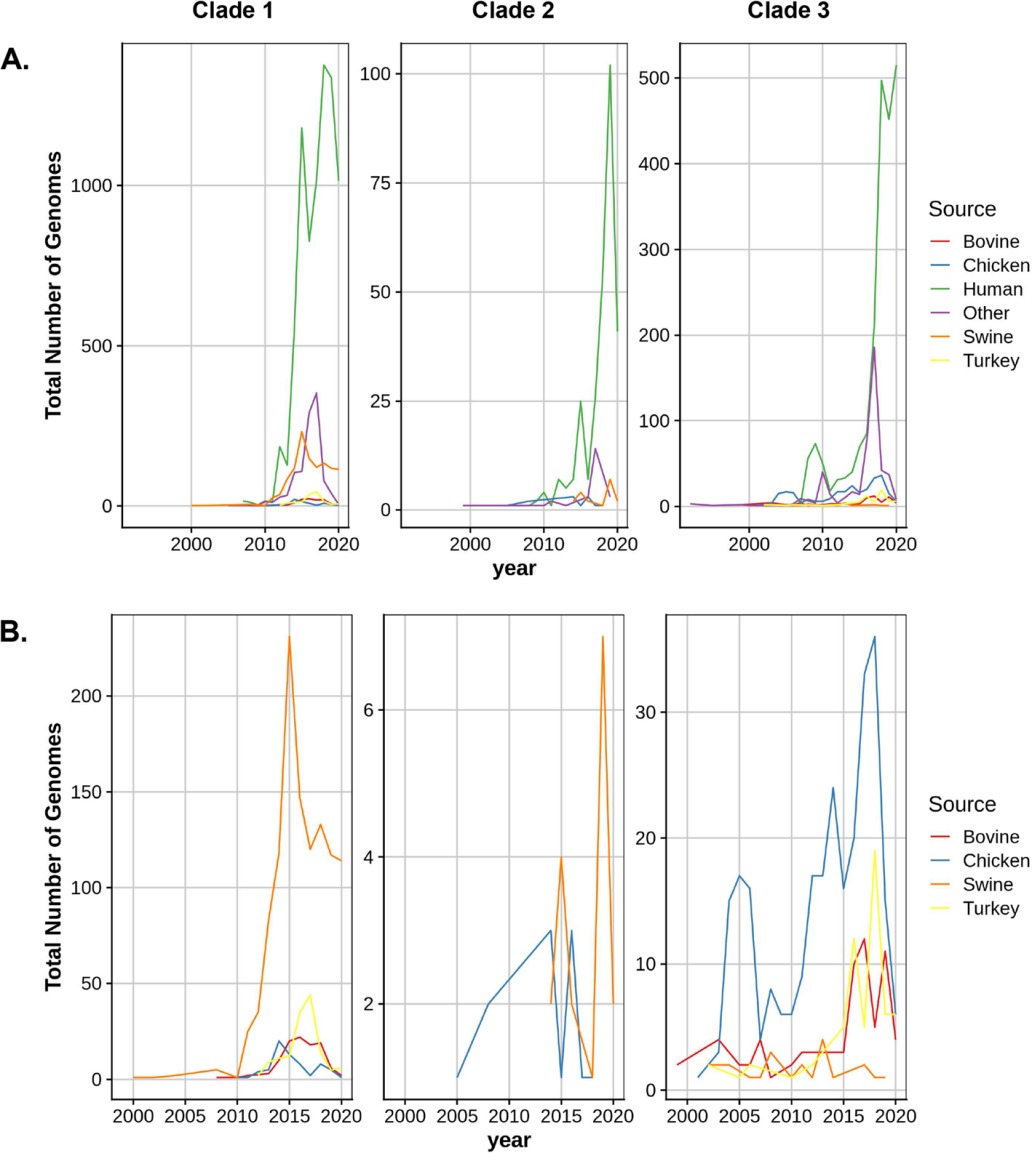
Isolation of serovar I 4,[5],12:i:- strain sequences in individual clades based on year. **A** Serovar I 4,[5],12:i:- strain sequence/source associations including human. **B** Association of serovar I 4,[5],12:i:- strain sequences with animal hosts. The scale of the y-axis varies between clades based on differences in the total number of genomes in each clade

Based on the metadata, 10,144 (75%), 1904 (14%), and 1564 (11%) serovar I 4,[5],12:i:- isolate sequences were associated with a human host, non-human host (chicken, bovine, swine, or turkey), or other, respectively, as shown on the second ring of the phylogenetic tree (Fig. 1) and depicted in Fig. 3A as the isolation rate over time for the individual clades. For isolate sequences associated with a non-human host, serovar I 4,[5],12:i:- was most commonly associated with swine (62%) followed by chicken (18%), turkey (11%), and bovine (9%) with the isolation rate over time for the individual clades illustrated in Fig. 3B. Serovar I 4,[5],12:i:- strains isolated from a specific host were more likely to be associated with an individual clade. For example, 96% of swine-associated strain sequences were included in clade 1 which contains 75% of the serovar I 4,[5],12:i:- isolates. Conversely, 77% of chicken-associated strain sequences were included in clade 3 which contains 22% of the serovar I 4,[5],12:i:- isolates. Swine- and chicken-derived isolates were significantly (< 0.0001) more likely to be associated with clades 1 and 3 compared to other animal-associated strains with an odds ratio of 38.24 (95% CI, 27.00 to 54.15) and 30.77 (95% CI, 22.80 to 41.53), respectively. The clade distribution of poultry-associated strain sequences differed based on genus; turkey-associated strain sequences were predominately included in clade 1 (69%) and slightly less than one-third (30%) were included in clade 3 with most of the chicken-associated isolates. The association of strains from the different phylogenetic clades with specific host species suggest certain genetic lineages of serovar I 4,[5],12:i:- strains may reside within a specific host.

### Accessory genome content and potential horizontal gene transfer dynamics

The accessory genome of this collection of isolates revealed some potential horizontal gene transmission (HGT) dynamics. Genes in the ‘shell’ partition were enriched for functions related to metal ion tolerance and detoxification, response to antibiotics, phage-related functions and others (Supplemental Table 3). The ‘cloud’ partition was enriched for a diverse set of functions, such as those related to facilitating horizontal gene transfer, responses to antibiotics, DNA integration, DNA recombination and phage activity (Supplemental Table 4). However, this pangenome partitioning may be biased due to clade 1 containing 75% of the genomes, as well as the observation that many of these isolates share numerous genes in common such as those present in SGI-4 (see below).

The ‘cloud’ partition of the pangenome, or accessory genome, linked isolates into clusters of shared gene content and these clusters were linked to phylogenetic placement as determined by the core genome alignment. We identified 3 clusters of accessory genome content. Genomes belonging to clade 1 mainly belonged to accessory genome cluster A (93% of clade 1 genomes). Genomes in clade 2 and 3 in general belonged to accessory genome cluster B (93 and 99% of clade 2 and 3 genomes respectively), while accessory genome cluster C was almost exclusively found in clade 1 (7% of clade 1 genomes, 0.9% of clade 2 genomes, 0.7% of clade 3 genomes) (Inner ring of Fig. 1). This separation between phylogenetic clades and accessory genome clusters suggests phylogenetic clades probably represent different populations that share accessory gene content within the clade but have not undergone extensive sharing of accessory gene content across different clades.

However, these results also suggest that HGT does exist across clades, either directly between *Salmonella* or via some intermediates. Some genomes in phylogenetic clade 1 belonged to accessory genome cluster B (0.251% of clade 1 genomes). Similarly, some clade 2 genomes belonged to accessory genome cluster A (6.41% of clade 2 genomes), and some genomes from clades 2 and 3 belonged to accessory genome cluster C (0.875 and 0.724% of clade 2 and 3 genomes respectively). The presence of shared accessory genome clusters across phylogenetic clades suggests some minor or low level gene flow between serovar I 4,[5],12:i:- isolates regardless of the core genome.

Plasmids, phages, and transposons were likely mediators of the accessory genome gene-flow that linked the different phylogenetic clades. GO term enrichment analysis suggested that the genes associated with accessory genome cluster A were phage, metal ion response, and type IV secretion related (Supplemental Table 5), accessory genome cluster B were pilus, conjugation, plasmid, and phage related (Supplemental Table 6), while terms associated with cluster C were related to conjugation and response to antibiotic (Supplemental Table 7). Previous work has demonstrated that phage transduction [15, 16] and plasmid conjugation [17–19] are common mechanisms for HGT of genes conferring phenotypic traits of interest such as antimicrobial resistance (AMR) and virulence. As more pathogen genomes become publicly available, it may be possible to more precisely interrogate the sources of these horizontally transferred genomic features.

### Prevalence of *Salmonella* genomic island 4 in serovar I 4,[5],12:i:- isolates

The earliest serovar I 4,[5],12:i:- strain sequence (L-3844) in the database containing an entire SGI-4 ICE was isolated in Japan in 2002, was a member of clade 1, and wasmost closely related to reference strain L-4234. Strain L-3844 was previously described by Aria et al. in their characterization of serovar I 4,[5],12:i:- emergence in Japan [14]. This group also described a serovar Typhimurium strain L-4126 isolated from bovine in 1998 that had both SGI-4 and the ASSuT phenotype.

**Fig. 4:**
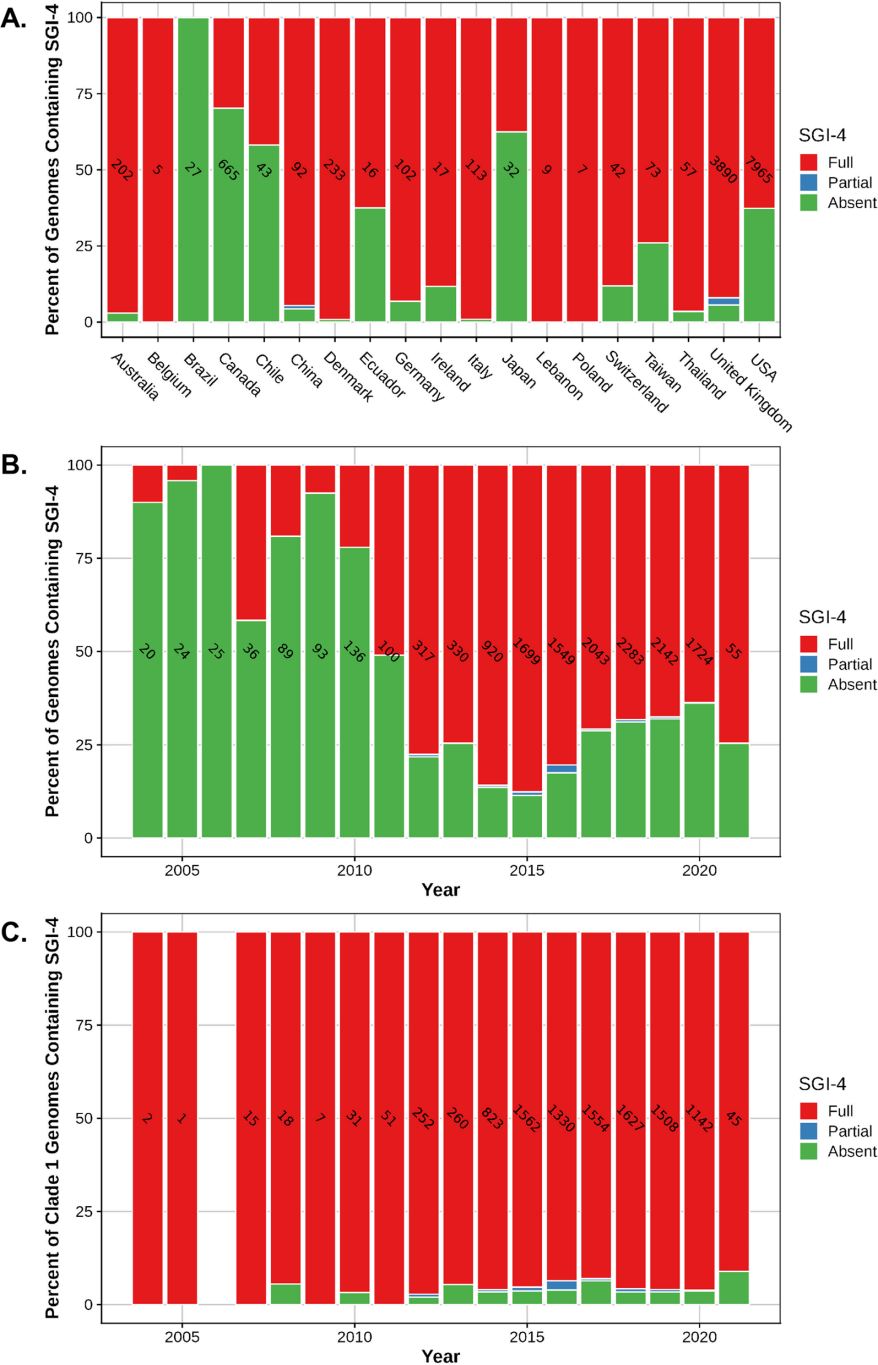
The presence of *Salmonella* genomic island 4 in serovar I 4,[5],12:i:- strain sequences. **A** The percent of all serovar I 4,[5],12:i:- strain sequences containing SGI-4 based on their country of origin. **B** The percent of all serovar I 4,[5],12:i:- strain sequences containing SGI-4 based on their year of isolation. **C** The percent of serovar I 4,[5],12:i:- strain sequences in clade 1 containing SGI-4 based on their year of isolation. The number of strain sequences from each country or for each isolation year is indicated within a bar

Interrogation of the serovar I 4,[5],12:i:- genomes demonstrated that the entire SGI-4 element containing genes for metal tolerance to copper, silver and arsenic was present in 71% of the 13,612 genomes and was almost exclusively present in isolates represented in clade 1, except for 1 strain (PNCS015114) isolated in 2012 from a human stool sample in Canada and associated with clade 3; 95% of clade 1 strains contained SGI-4. Clade 1 sequences most closely related to the serovar I 4,[5],12:i:- reference strains (L-4234, TW-Stm6, USDA15WA-1, and SO4698–09) contained SGI-4 at a frequency of 94–97%, indicating that the ICE containing the HMTGs was present in similar proportions for isolates related to these reference strains. In the entire dataset, the percentage of human-associated isolates containing SGI-4 was 73%, whereas 93, 57, 40, and 15% of the isolates associated with swine, bovine, turkey, and chicken harbored SGI-4, respectively. Swine-associated strain sequences were significantly (*P* < 0.0001) more likely to contain SGI-4 compared to all other animal-associated isolate sequences with an odds ratio of 29.50 (95% CI, 22.35 to 38.93). This finding supports other investigations in both Europe and the U.S. that have suggested an association between swine and serovar I 4,[5],12:i:- isolates containing SGI-4 [12, 20]. The strain sequences containing SGI-4 were globally distributed and isolated in all countries represented in the dataset except for Brazil, Guyana, and Trinidad and Tobago, however very few isolates originated from these countries; Fig. 4A indicates the percent of serovar I 4,[5],12:i:- strain sequences from each country that contain SGI-4. Although the overall representation of serovar I 4,[5],12:i:- isolate sequences in the dataset is clearly lower prior to 2014, a trend towards an increasing prevalence of genomes carrying SGI-4 is observed beginning in 2007 based on the number of sequences for each year (Fig. 4B). Analysis of strain sequences in clade 1 indicates the presence of SGI-4 in a high percentage (91–100%) of strains in 2004 to present day (Fig. 4C). Over time, clade 1 sequences became more prominent as a proportion of the total number of serovar I 4,[5],12:i:- strains (Fig. 2), but the prevalence of SGI-4 remained relatively constant for members of clade 1 (Fig. 4C). Therefore, the data indicate that SGI-4 was already present in a majority of strain sequences associated with clade 1 in 2004 and onward, suggesting that the presence of the SGI-4 ICE may have been instrumental for the successful emergence of serovar I 4,[5],12:i:- over the last two decades. Mastrorilli et al. [12] investigated 50 serovar I 4,[5],12:i:- strains collected between 2010 and 2016 in Italy and demonstrated that strains isolated after 2011 had a significantly higher prevalence of HMTGs compared to strains prior to that date. The authors suggested that the recent increase in acquisition of metal tolerance genes in serovar I 4,[5],12:i:- strains was potentially due to selective pressure exerted by the use of metals as alternatives to antimicrobials in animal husbandry, including swine production.

### Prevalence of the MDR module in serovar I 4,[5],12:i:- isolates

The earliest serovar I 4,[5],12:i:- strains in the NCBI Pathogen Detection database with an entire MDR module were MC_07–0902, MC_07–0968, N07–563, N07–620, N07–851, and VA_WGS-00174 and were isolated in 2007. Strain VA_WGS-00174 was associated with clade 3, was isolated from an environmental sample from a horse in Virginia (U.S.), and was most closely related to reference strain SL1344. Strains MC_07–0902, MC_07–0968, N07–563, N07–620, and N07–851 were isolated from human specimens, were members of Clade 1, and most closely related to reference strain TW-Stm6. Strains MC_07–0902 and MC_07–0968 were isolated in Ireland, and strains N07–563, N07–620, and N07–851 were isolated in Switzerland. These human-associated serovar I 4,[5],12:i:- strains contained both the MDR module and SGI-4, indicating that these two genetic elements were present in isolates circulating in human/animal populations prior to 2007.

**Fig. 5:**
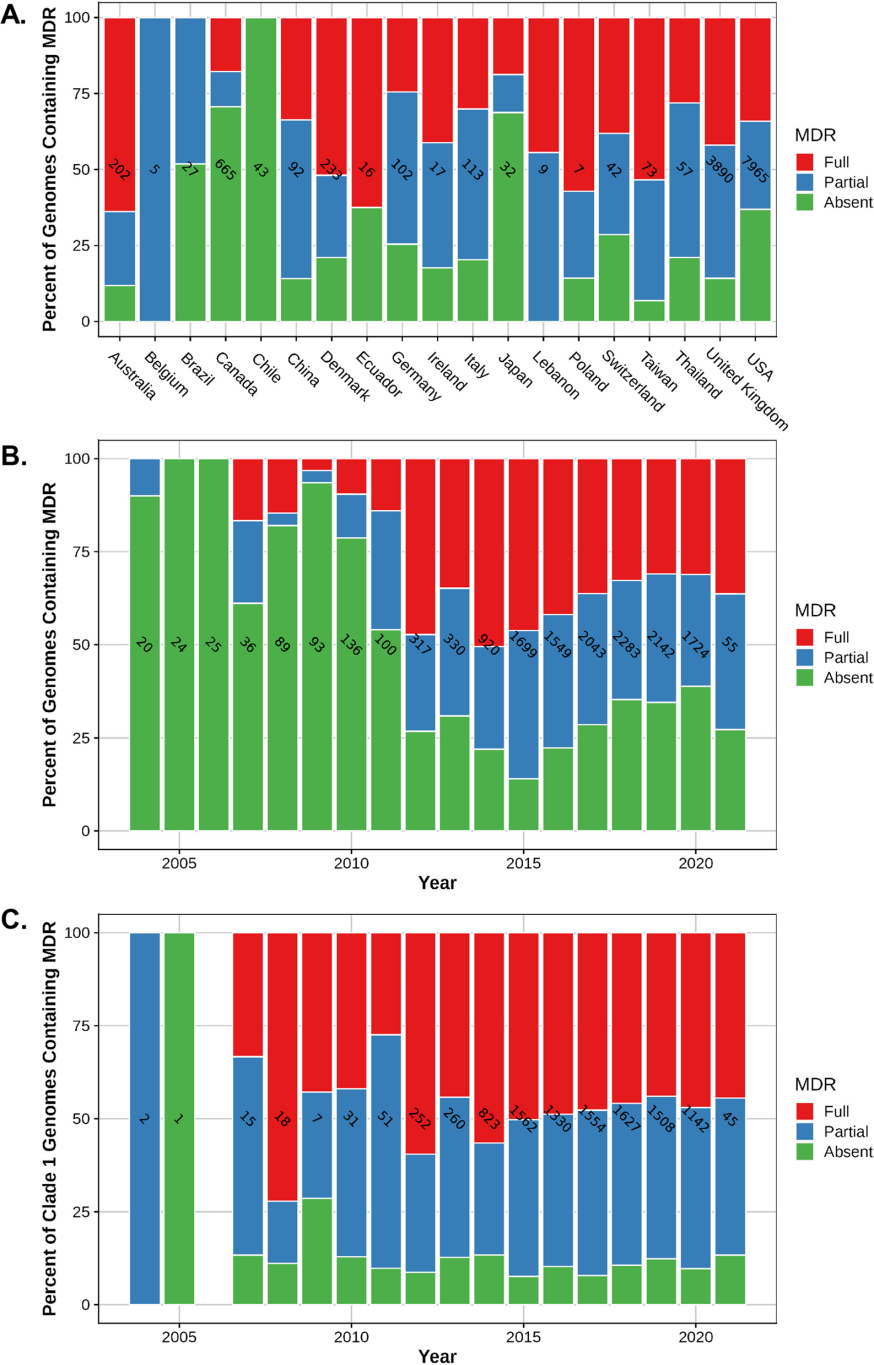
The presence of the MDR module in serovar I 4,[5],12:i:- strain sequences. **A.** The percent of all serovar I 4,[5],12:i:- strain sequences containing the MDR module based on their year of isolation. **B.** The percent of all serovar I 4,[5],12:i:- strain sequences containing the MDR module based on their country of origin. **C.** The percent of serovar I 4,[5],12:i:- strain sequences in clade 1 containing the MDR module based on their year of isolation. The number of strain sequences from each country or for each isolation year is indicated within a bar

The entire MDR module (R-type ASSuT with Hg tolerance) was present in 36% of the serovar I 4,[5],12:i:- genome sequences in the dataset and these strain sequences were almost exclusively associated with clade 1 (4920 isolates) except for strain VA_WGS-00174 described above. The average prevalence of the entire MDR module in isolate sequences most closely related to serovar I 4,[5],12:i:- reference strains TW-Stm6, USDA15WA-1, and SO4698–09 was 53%, but the frequency was only 24% in sequences most closely related to L-4234. Isolate sequences related to serovar I 4,[5],12:i:- reference strain L-4234 were significantly less likely (*P* < 0.0001) to contain the entire MDR module compared to sequences related to the reference strains TW-Stm6, USDA15WA-1, and SO4698–09 with an odds ratio of 0.2864 (95% CI, 0.2546 to 0.3223). Similar to SGI-4, the MDR module was also globally distributed in strain sequences from North America (U.S., Canada, and Mexico), Europe (United Kingdom, Denmark, Italy, Germany, Switzerland, Ireland, Poland and Sweden), Oceania (Australia), Asia (Taiwan, China, Thailand, Japan, Lebanon, Laos, Cambodia, and Vietnam), and South America (Ecuador); Fig. 5A indicates the percentage of serovar I 4,[5],12:i:- strain sequences containing the MDR module by country. Figure 5B illustrates an increase in the prevalence of the MDR module in serovar I 4,[5],12:i:- strain sequences after 2011. The prevalence of the MDR module in strain sequences associated with clade 1 was 40% from 2007 to 2011 (Fig. 5C); during this time frame, only 124 serovar I 4,[5],12:i:- isolate sequences were associated with clade 1. The prevalence of the MDR module in all strain sequences associated with clade 1 was 48%, indicating that although the number of sequences associated with clade 1 greatly expanded over time as a proportion of all serovar I 4,[5],12:i:- isolate sequences in the dataset (Fig. 2), the prevalence of the MDR module in clade 1-associated strain sequences remained relatively stable over time (Fig. 5C). The percentage of serovar I 4,[5],12:i:- strain sequences containing the MDR module in human-associated isolates was 38%, and for food animal-associated sequences was 39, 32, 29, and 7% from swine, bovine, turkey, and chicken, respectively. Swine-associated strain sequences were significantly (*P* < 0.0001) more likely to contain the MDR module compared to all other animal-associated sequences with an odds ratio of 2.813 (95% CI, 2.258 to 3.503).

Our analysis of the MDR module was based on the presence of the entire genetic region from strain USDA15WA-1. However, the MDR module is a mosaic of genetic elements from multiple sources and has several insertion sequences that may complicate DNA assembly [7]. Our DNA assemblies were derived from nucleotide sequences in the NCBI Pathogen Detection database that are frequently short read sequences, and these assemblies can result in numerous contigs instead of a single closed genome. In some of our genome assemblies, the break between contigs occurred in the MDR module due to its mosaic nature. Therefore, we also analyzed serovar I 4,[5],12:i:- genome sequences by screening for the presence of the antimicrobial resistance and heavy metal tolerance genes (*bla*_TEM − 1_, *strA* (*aph* (3″)-Ib), *strB* (*aph* (6)-Id), *sul2*, *tet*(B), and *merACDEPRT*) that are present in the MDR modules of USDA15WA-1 and the other serovar I 4,[5],12:i:- reference strains. The 12 AMR/HMT genes from the MDR module were present in 49% of serovar I 4,[5],12:i:- strain sequences in the dataset with 99% (6646) of the sequences belonging to clade 1; the remaining 1% of strain sequences containing all 12 AMR/HMT genes were from clades 2 (86) and 3 (5). The percentage of serovar I 4,[5],12:i:- strain sequences containing the 12 AMR/HMT genes from the MDR module was 51% for human-associated sequences, and 58, 41, 41, and 10% from swine-, bovine-, turkey-, and chicken-associated sequences, respectively. Similar to the analysis for the entire MDR module, swine-associated strain sequences were significantly (*P* < 0.0001) more likely to contain the 12 AMR/HMT genes compared to all other animal-associated sequences with an odds ratio of 3.896 (95% CI, 3.182 to 4.771).

Our analysis indicates that 36–49% of serovar I 4,[5],12:i:- strain sequences in the dataset contained the MDR module or all 12 genes for ASSuTHg. This difference is due to two separate methods to determine the presence of the module/genes. A conservative sequence analysis method required the presence of the MDR module and its submodules in the same configuration as found in USDA15WA-1, but this analysis most likely underestimated the presence of the module due to the fragmented nature of the assemblies, or the presence of the MDR submodules in difference genomic locations or configurations. An alternative analysis that screened for the presence of 5 AMR and 7 mercury tolerance genes identified a greater number of strain sequences compared to the conservative method, however, it is possible that the submodules of the MDR module were not present in the same configuration as identified in USDA15WA-1. The 12 AMR/HMT gene analysis better represents the presence of the genes of interest associated with the MDR module but does not guarantee the components of this genetic feature are arranged in the same manner as they are in strain USDA15WA-1.

### Co-occurrence of SGI-4 and the MDR module in serovar I 4,[5],12:i:- isolates

The earliest co-occurrence of SGI-4 and the MDR module in the NCBI Pathogen Detection database was described above for strains MC_07–0902, MC_07–0968, N07–563, N07–620, and N07–851 isolated in 2007. Not present in the NCBI Pathogen Detection database, serovar I 4,[5],12:i:- strain S0344705 was isolated in 2005, contained SGI-4, and conferred the ASSuT phenotype [8]. Furthermore, as indicated above, Aria et al. [14] described serovar Typhimurium strain L-4126 isolated in 1998 that had SGI-4 and the ASSuT phenotype; the insertion site for the MDR module, which typically inserts into the *fljB* region and converts serovar Typhimurium to serovar I 4,[5],12:i:-, was not described for strain L-4126.

**Fig. 6:**
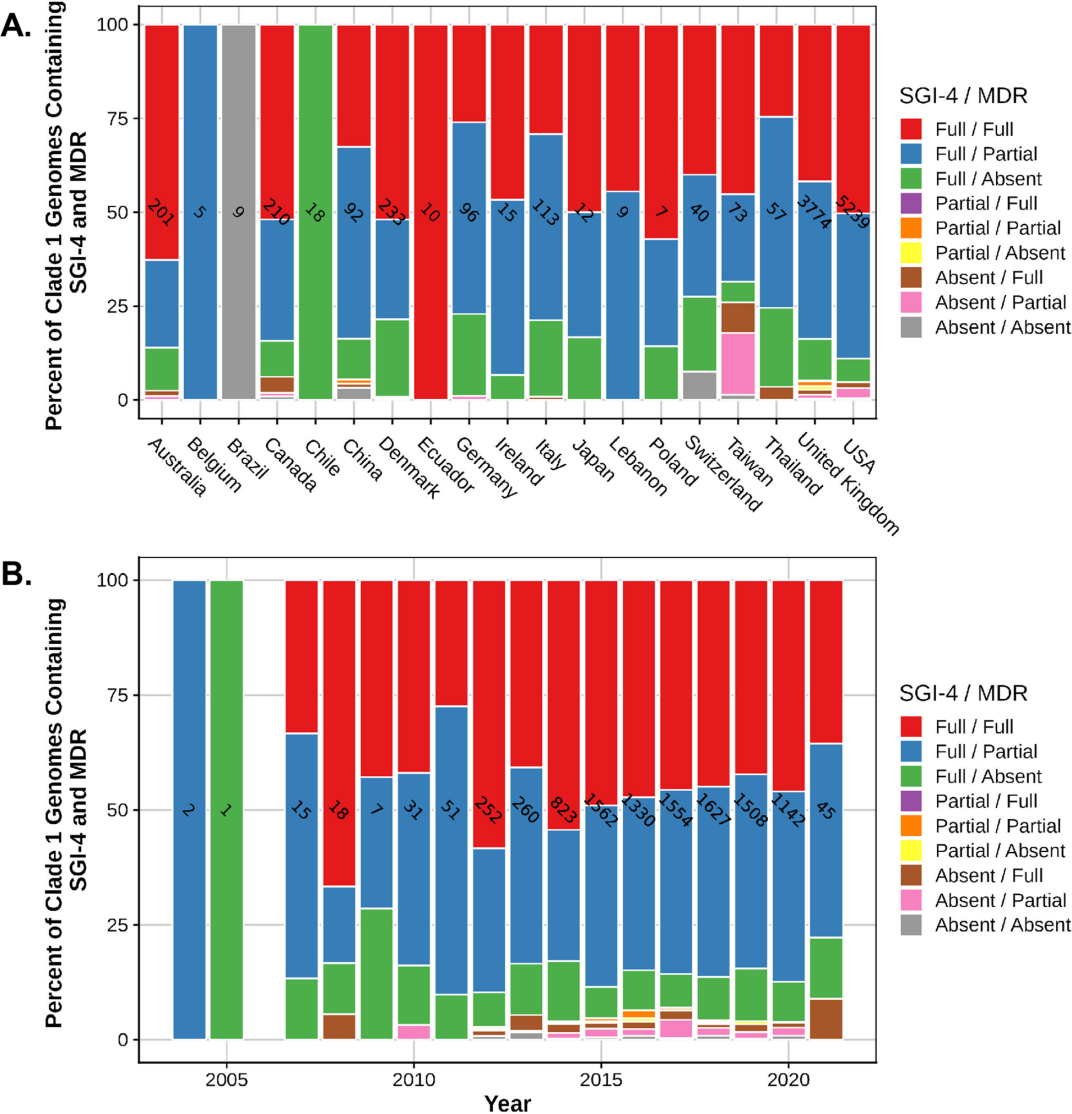
The presence of SGI-4 and the MDR module in serovar I 4,[5],12:i:- strain sequences. **A** The percent of serovar I 4,[5],12:i:- clade 1 members containing both SGI-4 and the MDR module based on the country of origin. **B** The percent of serovar I 4,[5],12:i:- clade 1 members containing both SGI-4 and the MDR module based on the year of isolation. The number of strain sequences from each country or for each isolation year is indicated within a bar

For the 13,612 serovar I 4,[5],12:i:- sequence dataset, 35% of genomes contain both SGI-4 and the MDR module, and all of these sequences were associated with clade 1. Figure 6A indicates the percentage of serovar I 4,[5],12:i:- strain sequences containing both SGI-4 and the MDR module by country. The co-occurrence of the MDR module and SGI-4 in strain sequences associated with clade 1 was 47%, and Fig. 6B indicates the percentage of clade 1-associated sequences containing both genetic elements over time. Only a subset of genomes (49%) containing SGI-4 also contained the MDR module, but most sequences (97%) containing the MDR module also contained SGI-4. The serovar I 4,[5],12:i:- sequences containing both the MDR module and SGI-4 mirror the data for genomes containing the MDR module individually (Fig. 5B), and these sequences displayed an increased prevalence of both genetic elements beginning in 2012 in relationship to the total number of serovar I 4,[5],12:i:- strains (data not shown). The percentage of serovar I 4,[5],12:i:- genomes containing both SGI-4 and the MDR module was 36% for human-associated sequences, and 39, 31, 23, and 6% for food animal-associated sequences from swine, bovine, turkey, and chicken, respectively. The co-occurrence of SGI-4 and the MDR module was similar between human and swine hosts. Swine-associated strain sequences were significantly (*P* < 0.0001) more likely to contain both SGI-4 and the MDR module compared to all other animal-associated sequences with an odds ratio of 3.136 (95% CI, 2.499 to 3.936).

Analysis of serovar I 4,[5],12:i:- strain sequences determined that 46% of isolates contained both SGI-4 and the 12 AMR/HMT genes from the MDR module. Forty-eight percent of human-associated strain sequences contained both SGI-4 and the AMR/HMT genes while 55, 40, 29, and 9% of swine-, bovine-, turkey-, and chicken-associated sequences contained these elements, respectively. For serovar I 4,[5],12:i:- genomes containing both SGI-4 and the complement of the 12 AMR/HMT genes, swine-associated sequences were significantly (*P* < 0.0001) more likely to contain these elements compared to all other animal-associated sequences with an odds ratio of 4.361 (95% CI, 3.535 to 5.380).

### Prevalence of *sopE* in serovar I 4,[5],12:i:- isolates

The genomes of *Salmonella enterica* isolates frequently contain prophage that contribute to strain diversity. These prophages usually insert into tRNA genes and can containing accessory genes including modulators of bacterial virulence. Petrovska et al. [8] described the monophasic *Salmonella *Typhimurium V (mTmV) prophage inserted in the *thrW* tRNA genomic region of serovar I 4,[5],12:i:- strain SO4698–09; the mTmV prophage in the SO4698–09 strain contains the *sopE* virulence gene encoding an effector that promotes pathogen invasion by interacting with the actin cytoskeleton of the mammalian host [21]. In their analysis, Petrovska et al. indicated that 30% of serovar I 4,[5],12:i:- strains isolated in the United Kingdom and Italy between 1993 and 2010 contained *sopE*. Furthermore, Palma et al. [22] described that 48% of European serovar I 4,[5],12:i:- strains isolated after 2010 contained *sopE*, and further demonstrated a negative association of *sopE* with serovar I 4,[5],12:i:- isolates from North America. Consistent with this finding, Elnekave et al. [20] indicated that only 5% of serovar I 4,[5],12:i:- strains isolated in the United States contained *sopE*. We interrogated the nucleotide sequences of serovar I 4,[5],12:i:- reference strains USDA15WA-1 and TW-STM6 for *sopE* and determined that this virulence gene is not present in either genome. Further investigation of the 13,612 serovar I 4,[5],12:i:- genomes from the NCBI dataset revealed that 2541 (19%) sequences contained *sopE* as depicted on the third ring from the outside on Fig. 1; sequences containing *sopE* were members of clades 1 (98%) and 3 (2%). The majority (76%) of clade 1 genomes containing *sopE* were from Europe. The remaining clade 1 sequences containing *sopE* were from North America (21%), Oceania (1%), and Asia (1%). Clade 1 genomes from Europe were significantly (*p* < 0.0001) more likely to contain *sopE* compared to sequences from either North America or all other continents (including North America) with an odds ratio of 7.462 (95% CI, 6.697 to 8.314) and 7.258 (95% CI, 6.540 to 8.056), respectively. This data supports the findings of Palma et al. [22] that *sopE* containing strains were more likely to be isolated in Europe compared to North America especially given that 64% of the serovar I 4,[5],12:i:- strain sequences from the NCBI dataset are from North America and 32% from Europe. In contrast to clade 1 strains, all serovar I 4,[5],12:i:- genomes containing *sopE* in clade 3 were from North America. Clade 1 strain sequences containing *sopE* were most frequently isolated from human (78%), swine (11%), and other (9%) sources and only isolated from turkey, chicken, and bovine sources at 1% each. The clade 3 strain sequences containing *sopE* were isolated from human (82%), chicken (11%), swine (5%), and bovine (2%) sources. The earliest strain in the NCBI dataset containing *sopE* was isolated in Switzerland from a human sample in 2007 (N07–1933). During the recent time period of 2014–2020, the prevalence of serovar I 4,[5],12:i:- strain sequences containing *sopE* as a subset of all genomes was greatest for years 2014–2016 (29%), decreased to 15% for years 2017–2019, and in 2020 was only 8%. The yearly prevalence of *sopE* in serovar I 4,[5],12:i:- genomes suggests that the presence of this virulence gene as a proportion of strain sequences is decreasing. Furthermore, the lower occurrence of *sopE* in serovar I 4,[5],12:i:- strains compared to genetic elements such as SGI-4 indicates that the overall role of this virulence determinant in successful emergence of this serovar was limited.

### Antimicrobial resistance differences between serovar I 4,[5],12:i:- clades

**Fig. 7:**
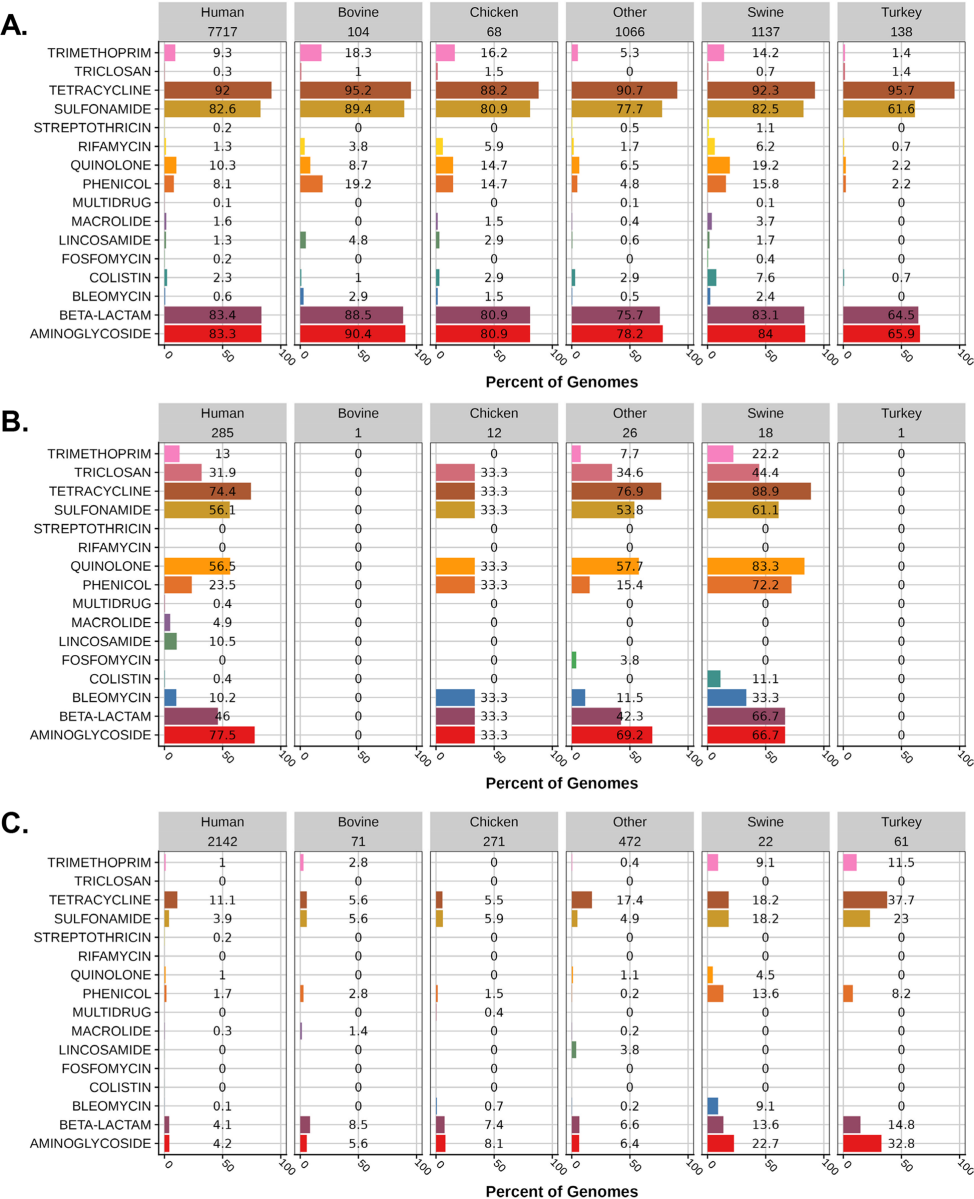
The presence of antimicrobial resistance genotypes in serovar I 4,[5],12:i:- strain sequences. **A** Percent of serovar I 4,[5],12:i:- clade 1 members containing antimicrobial resistance genotypes. **B** Percent of serovar I 4,[5],12:i:- clade 2 members containing antimicrobial resistance genotypes. **C** Percent of serovar I 4,[5],12:i:- clade 3 members containing antimicrobial resistance genotypes. The isolation source and the number of strain sequences for a given source are indicated at the top of each panel

Interrogation of the serovar I 4,[5],12:i:- genome sequences from the NCBI database indicated that 80% contain at least 1 antimicrobial resistance gene and 65% were classified as multidrug-resistant isolates. The percentage of serovar I 4,[5],12:i:- strain sequences containing resistance genes for at least 1 antimicrobial class (AMR) in human-associated isolates was 80%, and for food animal-associated sequences was 97, 85, 64, and 32% from swine, turkey, bovine, and chicken, respectively. Sixty-six percent of human-associated strain sequences contained genes for ≥3 antimicrobial classes (MDR) while 83, 55, 51, and 20% of swine-, bovine-, turkey-, and chicken-associated sequences contained genes for a minimum number of antimicrobial classes to be considered MDR. Swine-associated strain sequences were significantly (< 0.0001) more likely to contain genes for AMR and MDR compared to other animal-associated strains with an odds ratio of 26.79 (95% CI, 18.64 to 38.49) and 8.218 (95% CI, 6.637 to 10.18), respectively. Resistance to tetracycline, aminoglycoside, beta-lactam, and sulfonamide were overwhelmingly the most common antimicrobial classes present in serovar I 4,[5],12:i:- genomes with a frequency of 74, 66, 64, and 64%, respectively; the presence of all other classes of antimicrobial resistance was 10% or below. Resistance to the quinolone and colistin antimicrobial classes was present at 10 and 2%, respectively. Figure 7A-C shows the percentage of serovar I 4,[5],12:i:- genomes in each clade based on isolation source with antimicrobial resistance for up to 16 antimicrobial classes. The prevalence of antimicrobial resistance genes differed substantially between clades. For example, the presence of antimicrobial resistance genes was dramatically lower in clade 3 strain sequences compared to clades 1 and 2. Approximately 18% of the strains in clade 3 were resistant to at least 1 antimicrobial class and 4% were MDR. The AMR and MDR status of genomes represented in clade 2 was 80 and 71%, respectively, while strain sequences in clade 1 had the highest prevalence of AMR and MDR with 98 and 83%, respectively. The combination of increased MDR prevalence and greater representation (75%) of serovar I 4,[5],12:i:- genomes in clade 1 of the dataset correlates with serovar I 4,[5],12:i:- being the most frequent nontyphoidal multidrug-resistant serovar in the U.S. over recent years (2013, 2015–2018) [3]. The clade 1 strain D6–10116 isolated in China in 2015 from a human patient with acute gastroenteritis contained genes for resistance to 13 antimicrobial classes. Clades 1, 2, and 3 contained 190, 51, and 44 antimicrobial resistance patterns (single or combination), respectively. The ASSuT R-type associated with the MDR module is encoded by 5 AMR genes (*bla*_TEM − 1_, *strA* (*aph* (3″)-Ib), *strB* (*aph* (6)-Id), *sul2*, *tet*(B)) and has been described in serovar I 4,[5],12:i:- reference strains USDA15WA-1, TW-STM6, L-4234, and SO4698–09 for clade 1. For the entire dataset, 55% of serovar I 4,[5],12:i:- strain sequences contained the 5 resistance genes for R-type ASSuT. Approximately 72% of genomes in clade 1 had the ASSuT genes with or without additional AMR loci, while 54% of clade 1 sequences only had the 5 genes encoding the ASSuT R-type. One hundred fourteen (33%) of the genomes in clade 2 and only 10 (0.3%) strain sequences in clade 3 had the ASSuT R-type. Insertion of the MDR module in USDA15WA-1, TW-STM6, L-4234, and SO4698–09 deleted *fljB* (and an adjacent region), accounting for the monophasic (i:-) phenotype. Although the MDR module inserted into the *fljB* region may be mosaic with the potential for expansion and contraction of antimicrobial resistance genes, the data stimulates inquiry as to the type of genetic insertion/deletion that is causing the monophasic phenotype in strains which lack the MDR module.

Due to the presence of the MDR module in many of the serovar I 4,[5],12:i:- genomes, the antimicrobial resistance gene *bla*_TEM − 1_ was the most frequent β-lactamase, but other β-lactamase genes were also identified in the dataset including potential β-lactamase genes of clinical concern such as *bla*_CMY-2_, *bla*_SHV-12_, and *bla*_CTX-M_. The *bla*_CMY-2_, *bla*_SHV-12_, and *bla*_CTX-M_ genes were present in 274 (2%), 144 (1%), and 197 (1.4%) serovar I 4,[5],12:i:- isolates, respectively. A small number of genomes in each clade contained the *bla*_CMY-2_ gene with clades 1, 2, and 3 having 194 (1.9%), 2 (0.6%), and 79 (2.6%) strain sequences harboring this β-lactamase, respectively. Genomes containing the *bla*_CMY-2_ gene in clade 1 were associated with human (49%), swine (34%), bovine (6%), turkey (5%), other (5%) and chicken (1%) sequences in the USA (68%), United Kingdom (23%), Taiwan (6%), Canada (2%), Australia (1%), and China (1%). Bovine-associated isolate sequences of serovar I 4,[5],12:i:- in clade 1 were significantly (*p* = 0.03) more likely to contain *bla*_CMY-2_ compared to all other animal-associated (swine, turkey, and chicken) sequences with an odds ratio of 2.145 (95% CI, 1.126 to 4.084). This analysis may appear counter-intuitive but 12% of bovine-associated isolate sequences in clade 1 contain *bla*_CMY-2_ compared to 7, 6, and 3% of genomes associated with turkey, swine, and chicken. Two clade 2 strains isolated from humans in the USA and United Kingdom contained the *bla*_CMY-2_ gene. Strain sequences containing the *bla*_CMY-2_ gene in clade 3 were isolated from human (64%), chicken (20%), bovine (6%), other (4%), turkey (3%), and swine (3%) sources in Canada (56%) and the USA (44%). All genomes containing the extended spectrum β-lactamase gene (ESBL) *bla*_SHV-12_ were members of clade 1, and these strains were isolated from human (53%), swine (35%), other (10%), bovine (1%), and turkey (1%) sources from the USA (87%), United Kingdom (8%), Germany (4%), and Mexico (1%); swine-associated isolate sequences of serovar I 4,[5],12:i:- in clade 1 were significantly (*p* = 0.0005) more likely to contain *bla*_SHV-12_ compared to all other food-producing animals (bovine, turkey, and chicken) with an odds ratio of 7.232 (95% CI, 1.750 to 29.88). Serovar I 4,[5],12:i:- genomes containing *bla*_CTX-M_ genes were members of clades 1 (167), 2 (1), and 3 (28). Strains containing *bla*_CTX-M_ genes were members of clade 1 and isolated from human (63%), swine (26%), other (7%), chicken (2%), and bovine (2%) sources in China (37%), USA (28%), United Kingdom (21%), Australia (4%), Canada (4%), Germany (3%), Thailand (2%), Brazil (1%), Denmark (1%), and Japan (1%). The clade 2 strain containing the *bla*_CTX-M-2_ gene was isolated in Germany from a source classified as other. Strains containing *bla*_CTX-M_ genes in clade 3 were isolated from other (68%), human (29%), and bovine (4%) sources in the USA (89%) and the United Kingdom (11%). This set of genomes contains 13 types of *bla*_CTX-M_ genes including *bla*_CTX-M-1_ (7), *bla*_CTX-M-2_ (1), *bla*_CTX-M-3_ (1), *bla*_CTX-M-8_ (1), *bla*_CTX-M-9_ (2), *bla*_CTX-M-14_ (28), *bla*_CTX-M-15_ (12), *bla*_CTX-M-27_ (1), *bla*_CTX-M-32_ (2), *bla*_CTX-M-55_ (95), *bla*_CTX-M-65_ (42), *bla*_CTX-M-104_ (1), and *bla*_CTX-M-115_ (6).

As indicated above, 10% (1333) of serovar I 4,[5],12:i:- sequences contained genes for decreased susceptibility to ciprofloxacin (quinolone antimicrobial class) and 90% (1203) of these isolates were MDR. Eighty percent of strain sequences containing genes for decreased susceptibility to ciprofloxacin (DSC) also contained genes for resistance to an additional 4 or more antimicrobial classes; 5% of DSC isolates contained 2 or more alleles for DSC. Twenty-four genes/gene variants or point mutations conferred DSC including *qnrB19* (55%), *qnrS1* (18%), *qnrB2* (8%), *aac (6′)-Ib-cr5* (2%), *oqxB* (1%), *oqxA* (1%), *oqxA2* (1%), *qnrB1* (1%), *oqxB2* (1%), *qnrB6* (0.4%), *qnrS2* (0.4%), *qnrD1* (0.3%), *qnrVC1* (0.3%), *qnrS* (0.2%), *qnrA1* (0.2%), *qnrB77* (0.2%), and *qnrB4* (0.1%). Point mutations in *gyrA* (S83F, S83Y, D87N, D87Y, D87G), *gyrB* (E466D), and *parE* (H462Y) were identified that would reduce susceptibility to quinolone antimicrobials.

In May 2021, the FDA issued a NARMS Retail Meat Testing Interim Data Update [23] for an MDR *Salmonella* I 4,[5],12:i:- strain (19MN11PC02) isolated from a pork chop in 2019 due to an extensive resistance profile (resistant to 10 of 14 antibiotics on the NARMS *Salmonella* panel) including genes for resistance to cephalosporin (*bla*_CTX-M-15_) and fluoroquinolone (*aac (6′)-Ib-cr5* and *qnrB1*). Strain 19MN11PC02 and an additional 10 strains are present in the serovar I 4,[5],12:i:- dataset with MDR profiles including the genes *bla*_CTX-M-15_, *aac (6′)-Ib-cr5*, and *qnrB1*; nine of the strains were isolated from human clinical samples and the remaining strain was isolated from another pork sample. All of the isolates with the extensive antimicrobial resistance profile were isolated in the U.S.

Antimicrobial resistance genes for colistin were present in 2% of serovar I 4,[5],12:i:- strain sequences with the prevalence of the following genes: *mcr-9.1* (59%), *mcr-1.1* (26%), *mcr-3.1* (8%), *mcr-9* (2%), *mcr-1* (1.7%), *mcr-3.2* (1.7%), *mcr-3.20* (1.3%), *mcr-3.21* (1%), *mcr-3.24* (1%), *mcr-3* (0.7%), and *mcr-4.6* (0.7%). Serovar I 4,[5],12:i:- strain sequences containing colistin resistance genes were almost exclusively (99%) members of clade 1 with only 1 and 0.3% of sequences from clades 2 and 3, respectively. Sixty percent of serovar I 4,[5],12:i:- strain sequences with colistin resistance genes or 1.3% of all isolates in the dataset also contained quinolone resistance genes.

### Potential transmission clusters and geographic distribution of closely related serovar I 4,[5],12:i:- isolates

**Fig. 8:**
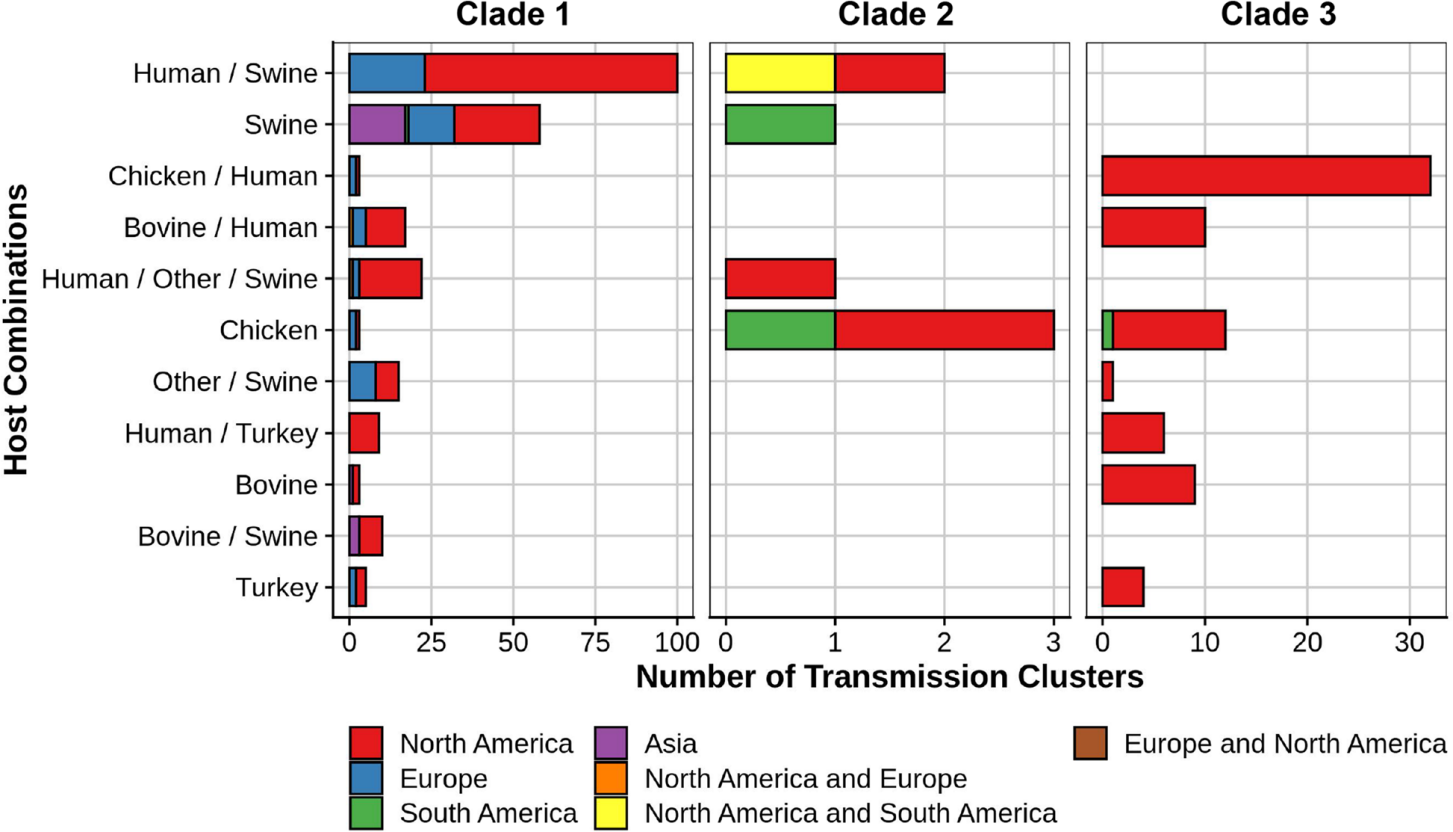
Serovar I 4,[5],12:i:- transmission clusters based on source(s) for clades 1, 2, and 3 colored by the geographic distribution of the transmission cluster. Transmission clusters are listed from greatest to fewer clusters per category from top to bottom. The transmission clusters human, human/other, and other are not included nor categories with fewer than 10 clusters

To determine potential transmission clusters of serovar I 4,[5],12:i:- strains between hosts, transmission clusters were inferred for closely related genomes. This analysis only identifies groups of very closely related isolate sequences and is not able to describe the precise route, mechanism, or directionality with which strains transfer between various sources and hosts. These results can inform future studies that aim to describe the route of transmission more precisely for *Salmonella* isolates of this type. Supplemental Table 8 illustrates the 1940 putative transmission clusters, and 1265 were classified as being sourced only from humans. However, human to human transmission, although certainly possible, is rare compared with transmission to humans from a non-human source [24]. The identification of transmission clusters only associated with humans is most likely due to a bias in the dataset because 75% of the serovar I 4,[5],12:i:- isolate sequences were derived from a human source, suggesting the need for additional surveillance sampling from non-human sources. Transmission clusters between human and “other” sources (177), and between “other” sources (99) were also identified (Supplemental Table 8). The “other” source designation includes environmental samples; thus, the transmission between human and other sources could represent closely related strains isolated from slaughter plant surveillance and human clinical cases. Additionally, the source designation of “other” is generally a catchall for samples with incomplete metadata. Figure 8 identifies the most frequent transmission clusters by source(s) (excluding human, human/other, and other transmission clusters) for individual clades that were within and across continents. The most frequent transmission clusters predicted for humans and a distinct primary source(s) was human/swine-associated (103), human/chicken-associated (36), human/bovine-associated (27), and human/other/swine-associated (24). The human/swine-associated and human/other/swine-associated transmission clusters were predominately represented by clade 1 sequences containing 101 and 22 clusters, respectively. In contrast, human/chicken-associated transmission clusters were predominately associated with clade 3 containing 33 clusters. The human/bovine-associated transmission cluster was split between clades 1 (17) and 3 (10). Transmission clusters only represented with an individual animal-associated source were also identified for swine- (61), chicken- (20), bovine- (12), and turkey- (10) associated sequences. As expected, based on their previously described clade memberships, transmission clusters containing only swine- (60) or chicken- (14) associated sequences were predominately affiliated with clades 1 and 3, respectively. Serovar I 4,[5],12:i:- strain transmission clusters linking two animal-associated hosts were also predicted including bovine/swine- (10), chicken/swine- (3), chicken/bovine- (1), and swine/turkey- (1) associated sequences. As clade 2 contains the fewest serovar I 4,[5],12:i:- isolate sequences in the dataset, it also contained the lowest number of transmission clusters (54).

Transmission clusters containing serovar I 4,[5],12:i:- strain sequences that were isolated in at least two different countries (146) were predicted in the transmission data including a subset between continents (91) (Supplemental Table 8). Transmission clusters containing sequences from both the USA and the United Kingdom (50) were the most frequent for both intercountry and intercontinental. As serovar I 4,[5],12:i:- strain sequences from the USA and the United Kingdom represent the greatest number of sequences in the dataset, the finding that certain strain sequences from these countries are more closely related is not unexpected. Transmission clusters for closely related strain sequences between the USA and Canada (29), the United Kingdom and Canada (11), the United Kingdom and Denmark (10), and the United Kingdom and Australia (9) were also observed as more common compared to other groups for intercountry transmission. Nine transmission clusters contained closely related strain sequences from three independent countries with the USA, the United Kingdom, and Canada being involved in 4 intercountry clusters. Transmission clusters containing serovar I 4,[5],12:i:- strain sequences from multiple countries suggests that closely related isolate sequences are present across geographic boundaries.

The second largest transmission cluster (phydelity cluster 2307) of closely related strain sequences contained 45 isolate sequences and was derived from a combination of human-, other-, and swine-associated sources. Cluster 2307 contains the nucleotide sequence for strain USDA15WA-1 (FSIS1503788) [6] isolated from swine cecal contents that was collected by USDA FSIS at the pork processing plant associated with the 2015 multi-state pork outbreak; eight additional isolates in cluster 2307 were also isolated by FSIS at the outbreak-associated pork processing plant. Eighteen clinical strains in cluster 2307 were identified during the investigation as being associated with the pork outbreak [25]. In addition to cluster 2307, 4 additional transmission clusters contained closely related isolate sequences associated with the pork outbreak based on strains described in Kawakami et al. [25] and involved clusters containing human/other/swine- (2489 and 842) or human-associated (2488 and 2077) strain sequences. These 5 transmission clusters, containing strain sequences associated with the 2015 pork outbreak, included a total of 94 isolate sequences with 53 (56%) outbreak-associated and 41 considered sporadic as they were not described in the outbreak investigation. Approximately half of the isolate sequences derived from human- and swine-associated sources in these clusters were linked to the outbreak, but 85% of strain sequences from the “other” source including environmental swabs were associated with the pork outbreak. Although the majority (79%) of strain sequences involved in these transmission clusters were isolated in 2015 and 2016 during the pork outbreak time period, 20 of the strains included in these transmission clusters were isolated between 2017 and 2020 indicating that isolates closely related to the 2015 pork outbreak strains continued to circulate and cause human illness. Of the 94 isolates from transmission clusters related to the 2015 U.S. pork outbreak, 1 strain was isolated from a human sample in the United Kingdom in 2019. The FSIS and human clinical isolates were originally linked to the pork outbreak based on their pulsed-field gel electrophoresis (PFGE) patterns that indicated strain relatedness. Identification of transmission clusters with numerous isolates previously associated with a known outbreak demonstrates agreement between PFGE and WGS (whole genome sequencing) cluster analysis and corroborates the use of WGS for detection of transmission clusters, as well as the continued circulation of closely related strains.

## Conclusion

Of the > 2600 *Salmonella* serovars, *Salmonella* serovar I 4,[5],12:i:- has emerged as a top 5 cause of human foodborne disease, and many of these strains have an ASSuT R-type encoded on an MDR module and an ICE (SGI-4) with multi-metal tolerance (CuAgAs). From 2014 to 2020, the average prevalence of SGI-4 in serovar I 4,[5],12:i:- isolate sequences was 71%. Although the presence of the MDR module in serovar I 4,[5],12:i:- isolates has increased over the last decade, the percentage of isolate sequences containing the MDR module is overall lower (36–49%) compared to those containing SGI-4 over the same time period. The data analysis by year suggests that the proportion of serovar I 4,[5],12:i:- isolate sequences containing SGI-4 and/or the MDR module was most likely increasing prior to 2014 but then remained relatively constant since 2014. However, definitive discussion of this increasing trend is tempered due to the smaller number of genome sequences present in the NCBI database prior to 2014.

Another trend identified in the 13,612 sequence dataset is increasing AMR over time, which is supported by NARMS designating serovar I 4,[5],12:i:- as the most frequent non-typhoidal MDR *Salmonella* serovar in the U.S. over recent years. From the dataset, 65% of strain sequences were classified as MDR, 5% of sequences contained β-lactamase genes of clinical concern (such as *bla*_CMY-2_, *bla*_SHV-12_, and *bla*_CTX-M_), and 10% of sequences contained genes for decreased susceptibility to the quinolone antimicrobial class. These AMR results are highlighted by a recent NARMS Retail Meat Testing Interim Data Update [23] issued by the FDA for an MDR *Salmonella* I 4,[5],12:i:- strain isolated from a pork chop with an extensive resistance profile including genes for resistance to cephalosporin (*bla*_CTX-M-15_) and fluoroquinolone (*aac (6′)-Ib-cr5* and *qnrB1*).

Due to multiple factors including continual improvements in high-throughput capacity and affordability for sequencing bacterial genomes, the NCBI Pathogen Detection dataset for the 13,612 serovar I 4,[5],12:i:- WGSs has a lower prevalence of strain sequences for the two decades spanning the 1990s and 2000s that limits data interpretation for the emergence of serovar I 4,[5],12:i:- strains including isolates containing SGI-4 and the MDR module. Molecular and computational technologies now permit genomic sequencing of pathogenic isolates from clinical, agricultural, and environmental sources, and is replacing previous methods for determination of bacterial genetic relatedness including pulsed-field gel electrophoresis. Therefore, the majority of serovar I 4,[5],12:i:- WGS in the NCBI Pathogen Detection database are derived from strains isolated between 2014 and 2020; this time frame also corresponds to the peak emergence of this serovar as a cause of human foodborne disease (4th most frequent *Salmonella* serovar in the U.S.), including the U.S. pork outbreak in 2015, and the rise of serovar I 4,[5],12:i:- as the most common MDR *Salmonella* serovar. In the serovar I 4,[5],12:i:- WGS dataset, swine-associated sequences were the most common food-animal source containing SGI-4, the MDR module, or both. Furthermore, transmission cluster analysis inferred that swine-associated sequences were frequently linked to closely related human-associated sequences, suggesting potential transmission dynamics for this serovar. This study illustrates how analysis of WGSs from the NCBI Pathogen Detection database can be utilized to identify the prevalence of genetic features that may be responsible for the successful emergence of foodborne pathogens.

## Methods

### Data acquisition and quality control

The metadata associated with NCBI Pathogen Detection database [26] version PDG000000002.2107, was downloaded on January 24, 2021. We identified PDS groups containing isolates with reported serotypes of “I 4,[5],12:i:-” and ‘monophasic Typhimurium’. We downloaded all genome assemblies from these PDS groups and predicted serotypes for all isolates using SeqSero2 [27]. All genomes typed as “I 4,[5],12:i:-” were included in further analyses. Highly fragmented assemblies and those with possible contamination (greater than 200 contigs and total assembly length > 5.7mb) were removed. The final collection contained 13,612 genomes. The dataset included 32 strains from Japan and 443 of 811 Canadian isolates that were previously described by Aria et al. and Clark et al., respectively [14, 28]. The broad category for source of isolate sequences was extracted from the ‘isolation_source’ and ‘host’ columns in the metadata (Supplemental Table 1) provided by the NCBI Pathogen Detection database. Any isolate sequences with the terms ‘human’, ‘homo’, or ‘sapiens’ were classified as ‘human-associated’ and denoted as ‘Human’. Any isolate sequences with the terms ‘swine’, ‘pork’, ‘porcine’, ‘sow’, ‘sus’, ‘scrofa’, ‘hog’, or ‘pig’, were classified as ‘swine-associated’ and denoted as ‘Swine’. Isolate sequences with the terms ‘bovine’, ‘beef’, ‘veal’, ‘cow’, ‘cattle’, ‘bos’, ‘taurus’, ‘steer’, or ‘calf’ were classified as ‘bovine-associated’ and denoted ‘Bovine’. Isolate sequences with the terms ‘chicken’, ‘chick’, ‘gallus’, ‘broiler’, or ‘egg’, were classified as ‘chicken-associated’ and denoted ‘Chicken’. Isolate sequences with the terms ‘turkey’, ‘meleagris’, or ‘gallopavo’ were classified as ‘turkey-associated’ and denoted ‘Turkey’. Isolate sequences with ‘isolation_source’ or ‘host’ values that were not classified as human-, swine-, bovine-, chicken-, or turkey-associated (as described above) were classified and denoted as ‘other’. Classical 7 gene multilocus sequence types (MLST) were calculated using the mlst tool [29, 30].

The 13,612 genomes in this set originated from North America [64% (United States (~ 92%; 7965), Canada (8%; 665), and Mexico (0.05%; 4))], Europe [32% (United Kingdom (~ 88%; 3890), Denmark (5%; 233), Italy (3%; 113), Germany (2%; 102), Switzerland (1%; 42), Ireland (0.4%; 17), Poland (0.2%; 7), Belgium (0.1%; 5), France (0.1%; 3), Spain (0.02%; 1), and Sweden (0.02%; 1))], Asia [2% (China (34%; 92), Taiwan (27%; 73), Thailand (21%; 57), Japan (12%; 32), Lebanon (3%; 9), Laos (1%; 3), Cambodia (0.7%; 2), and Vietnam (0.4%; 1))], Oceania [1% (Australia (100%; 202))], and South America [1% (Chile (46%; 43), Brazil (29%; 27), Ecuador (17%, 16), Guyana (3%; 3), Colombia (2%; 2), Barbados (1%; 1), and Trinidad and Tobago (1%; 1))]. The number of serovar I 4,[5],12:i:- strain sequences in the NCBI Pathogen Detection database increased over multiple years, peaking in 2018 (2288) (Supplemental Fig. 1). The earliest serovar I 4,[5],12:i:- strain sequence (PDT000149207.2) in the database was collected in June 1992 from equine feces in Oklahoma (USA).

### Biases and limitations in the available data

As the data available in the NCBI Pathogen Detection database are composed of publicly deposited sequences, and not all countries have equivalent *Salmonella* sequencing efforts or utilize this database for routine deposition of sequences, isolate sequences from a few countries make up the majority of the sequences in this collection. Most notably, isolates from the United States (7965 isolates) and the United Kingdom (3890 isolates) represent a large majority of the genome sequences. It should not be concluded that serovar I 4,[5],12:i:-, certain genetic attributes, or antimicrobial resistance (AMR)/MDR profiles are necessarily more prevalent in these countries, nor that this serovar is lower or absent from other countries including those not represented in this database, but rather these numbers are reflective of larger pathogen sequencing efforts or the use of this database by institutions in the United States and the United Kingdom. Different patterns of strain distribution may become evident as more countries expand their pathogen sequencing efforts. Nonetheless this database is the current best resource for examining the distribution and characteristics of known *Salmonella* serovar I 4,[5],12:i:- strains. Strain sequences deposited in the NCBI Pathogen Detection database are derived from a combination of active surveillance and non-surveillance investigations from a variety of sample sources and institutions. Our data analysis did not separate sequences based on the source of samples (i.e. HAACP sampling) or submitting institutions, and therefore this analysis should not be used in the absence of additional context for regulatory decisions.

### BLAST searches for genomic features of interest

The genome assemblies were searched for full and partial segments of both SGI-4 (nucleotides 4,659,019–4,739,704) and the MDR module (nucleotides 2,916,967–2,945,159) from serovar I 4,[5],12:i:- strain USDA15WA-1 (NCBI accession CP040686), and the *sopE* gene (nucleotides 5,025,398–5,026,120) from serovar I 4,[5],12:i:- strain SO4698–09 (NCBI accession LN999997) via BLASTn using the default parameters. Because the majority of these genomes are fragmented short read only draft genomes, genomic sequences of interest sometimes were interrupted by contig breaks. In particular, the MDR module often prevented successful assembly of contigs (contig boundaries occurred within the sequence of the MDR module). Therefore, the presence of these features was determined by the coverage of the sequence of interest by BLAST hits in each genome considering only the top 3 high scoring pairs (minimum 95% identity). The percent of the sequence of interest covered determined if it was fully present 90–100%, partially present 50–89%, or absent 0–49%.

### Pangenome construction

All genomes were annotated with prokka [31], using proteins from *S. enterica* serovar Typhimurium strain LT2 (accession GCA_000006945.2) as a first priority, and a pangenome was constructed and partitioned using ppanggolin [32]. Ppanggolin uses statistical models to divide the pangenome into 3 classes: persistent genome, representing the gene families present in almost all genomes, the shell genome representing gene families present at intermediate frequencies in the species, and the cloud genome representing gene families present at low frequency in the species. Interproscan [33] was used to assign gene ontology (GO) terms against representative proteins.

### Clustering based on accessory genome content

To identify clusters of genomes with similar accessory gene content, the genes belonging to the ‘cloud’ partition were used to construct a co-occurrence matrix and this matrix was used to generate a weighted graph where the nodes are genomes, and the edges represent the number of shared cloud partition genes shared between genomes. This graph was subjected to the leiden [34] community detection algorithm (using the default parameters) to identify densely connected sub-communities. These ‘accessory genome clusters’ represent groups of isolates that contain similar accessory gene content. We then sought to determine which genes were enriched in each cluster. A gene was considered to be enriched in a cluster if the within-cluster proportion presence divided by the average proportion presence of the other two clusters was 2 or greater.

### GO term enrichment

To determine GO terms enriched in pangenome partitions, as well as those enriched in accessory genome clusters, the R package topGO [35, 36] was used. Gene sets composed of genes within each partition, as well as genes enriched within each accessory genome cluster were subjected to the ‘elim’ algorithm utilizing a fisher test statistic, GO terms with *P* values < 0.05 were considered significantly enriched.

### Core genome phylogenetic tree construction

To assess the phylogenetic relatedness of the isolates, we constructed a phylogenetic tree based on an alignment of the core genome. To ease the computational burden as well as assist in visualization, genomes were grouped by 1) continent or origin, 2) year of isolation, 3) isolation source (host animal), 4) `SNP cluster` (as assigned by NCBI Pathogen Detection pipeline), 5) accessory genome cluster (from ppanggolin pangenome). This produced 912 groups and a representative genome was chosen at random from within each. To this collection we added the reference genomes *S. enterica* serovar Typhimurium strains LT2 (GCA_000006945.2) and SL1344 (GCA_000210855.2), as well as *S. enterica* serovar Enteriditis strain 92–0392 (GCA_002761055.1). The core genes from these genomes were aligned with mafft [37] using the pipeline in Roary [38] using the default parameters. This core genome alignment was used to calculate a maximum likelihood phylogenetic tree using RAxML [39] implementing a GTR + G model. The resulting tree was used to calculate all pairwise cophenetic distances (branch lengths between all pairs of leaves), these distances were used to assign each representative genome to a ‘closest reference genome’ by assigning the reference genome with the lowest distance. The R [40] packages ape [41], ggtree [42] and tidyverse [43] were used for data wrangling, phylogenetic manipulations, and visualizations. GNU parallel [44] was used for parallel processing.

### Transmission cluster inference

The program Phydelity [45] was used to infer putative clusters of transmission. For each SNP cluster, the SNP based phylogenetic tree provided by NCBI Pathogen Detection was downloaded, SNP clusters with 5 or fewer isolates were excluded from this analysis. The remaining SNP trees were used as inputs to Phydelity with the default settings. In this usage, putative transmission clusters contain very closely related isolate sequences implying a common source.

### Statistical analysis

The Fisher’s exact test with a calculation of an odds ratio and a 95% confidence interval (CI) was performed in GraphPad Prism 5.01 (GraphPad Software, Inc., La Jolla, CA). On datasets with large sample numbers, GraphPad Prism automatically calculated a Chi-square test instead of a Fisher’s exact test. *P*-values less than 0.05 were considered significant.

## Supplementary Information

**Additional file 1: Figure S1.** The number of serovar I 4,[5],12:i:- strain sequences based on their year of isolation.

**Additional file 2: Table S1.** Metadata for 13,612 *Salmonella* serovar I 4,[5],12:i:- strain sequences from the NCBI Pathogen Detection database.

**Additional file 3: Table S2.** The number (%) of *Salmonella enterica* serovar I 4,[5],12:i:- strain sequences based on country of origin, closest reference strain, and clade membership.

**Additional file 4: Table S3.** *Salmonella* serovar I 4,[5],12:i:- genes in the shell genome representing gene families present at intermediate frequencies.

**Additional file 5: Table S4.** *Salmonella* serovar I 4,[5],12:i:- genes in the cloud genome representing gene families present at low frequencies.

**Additional file 6: Table S5.** GO term enrichment analysis for accessory genome cluster A.

**Additional file 7: Table S6.** GO term enrichment analysis for accessory genome cluster B.

**Additional file 8: Table S7.** GO term enrichment analysis for accessory genome cluster C.

**Additional file 9: Table S8.** Putative transmission clusters for *Salmonella enterica* serovar I 4,[5],12:i:- strain sequences.

## Data Availability

All metadata and accession numbers for genome assemblies in the NCBI database are available in Supplemental Table 1.
