# Supplementary material for: Relationship and distribution of Salmonella enterica serovar I 4,[5],12:i:- strain sequences in the NCBI Pathogen Detection database
Source: BMC Genomics. 2022 Apr 6;23:268. doi: 10.1186/s12864-022-08458-z (PMC8985322; doi:10.1186/s12864-022-08458-z)
Supplement: Supplementary file 1 — Additional file 1: Figure S1. The number of serovar I 4,[5],12:i:- strain sequences based on their year of isolation. [file 12864_2022_8458_MOESM1_ESM.pdf]

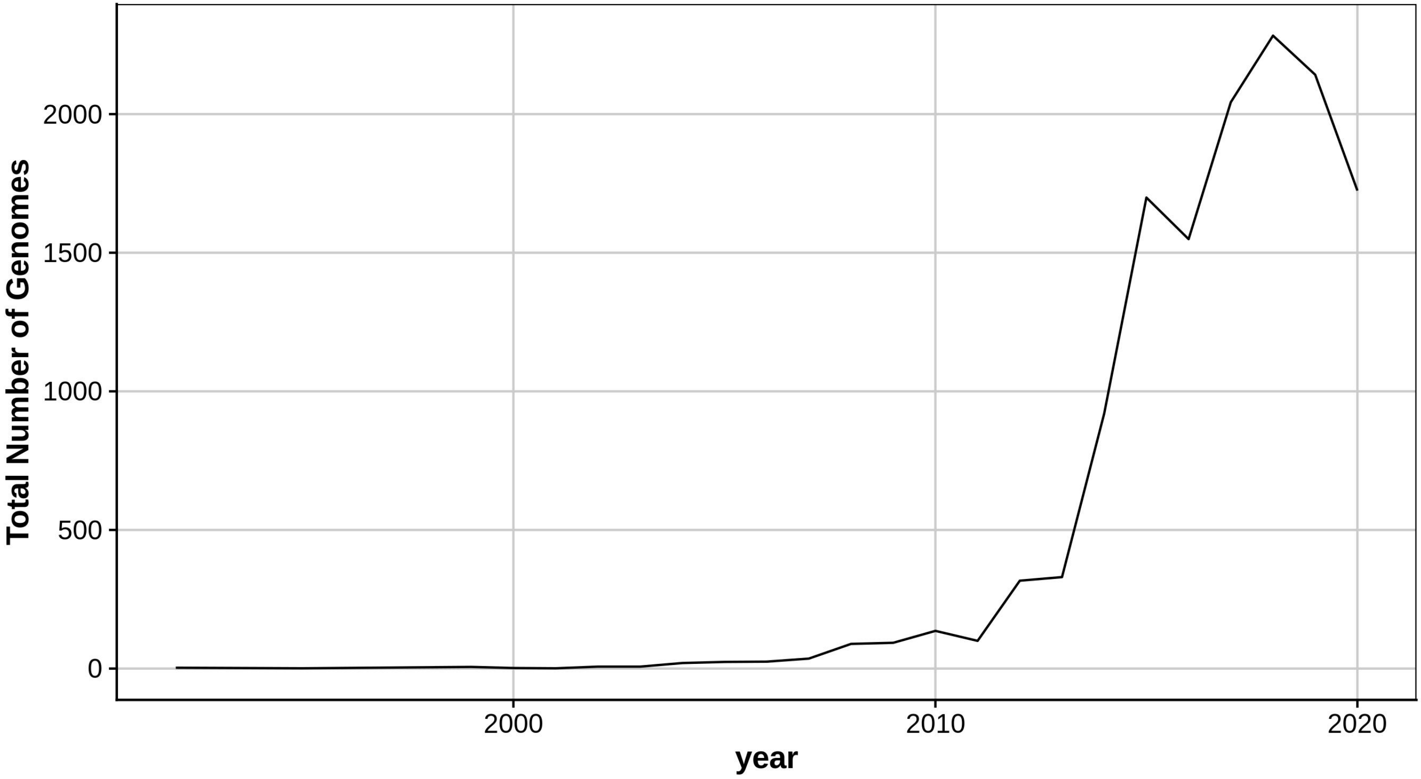

Supplemental Figure 1. The number of serovar I 4,[5],12:i:- strain sequences based on their year of isolation.
