# Supplementary material for: Relationship and distribution of Salmonella enterica serovar I 4,[5],12:i:- strain sequences in the NCBI Pathogen Detection database
Source: BMC Genomics. 2022 Apr 6;23:268. doi: 10.1186/s12864-022-08458-z (PMC8985322; doi:10.1186/s12864-022-08458-z)
Supplement: Supplementary file 3 — Additional file 3: Table S2. The number (%) of Salmonella enterica serovar I 4,[5],12:i:- strain sequences based on country of origin, closest reference strain, and clade membership. [file 12864_2022_8458_MOESM3_ESM.pdf]

Supplemental Table 2. The number (%) of *Salmonella enterica* serovar I 4,[5],12:i:- strain sequences based on country of origin, closest reference strain, and clade membership

			**Clade 1** 10230 (75.15)								**Clade 2** 343 (2.52)		**Clade 3** 3039 (22.33)	
	**Total**		**TW-Stm6** 3722	(36.38)	**USDA15WA-1** 3423	(33.46)	**L-4234** 1725	(16.86)	**SO4698-09** 1360	(13.29)	**LT2** 343	(100)	**SL1344** 3039	(100)
**North America**	8634	(63.43)	1875	(50.38)	3421	(99.94)	156	(9.04)			264	(76.97)	2918	(96.02)
USA	7965	(92.25)	1831	(97.65)	3282	(95.94)	126	(80.77)			240	(90.91)	2486	(85.20)
Canada	665	(7.70)	43	(2.29)	137	(4.00)	30	(19.23)			24	(9.09)	431	(14.77)
Mexico	4	(0.05)	1	(0.05)	2	(0.06)							1	(0.03)
**Europe**	4414	(32.43)	1427	(38.34)			1510	(87.54)	1351	(99.34)	55	(16.03)	71	(2.34)
United Kingdom	3890	(88.13)	1226	(85.91)			1299	(86.03)	1249	(92.45)	53	(96.36)	63	(88.73)
Denmark	233	(5.28)	82	(5.75)			82	(5.43)	69	(5.11)				
Italy	113	(2.56)	43	(3.01)			61	(4.04)	9	(0.67)				
Germany	102	(2.31)	41	(2.87)			37	(2.45)	18	(1.33)	1	(1.82)	5	(7.04)
Switzerland	42	(0.95)	27	(1.89)			8	(0.53)	5	(0.37)	1	(1.82)	1	(1.41)
Ireland	17	(0.39)	4	(0.28)			11	(0.73)					2	(2.82)
Poland	7	(0.16)	2	(0.14)			5	(0.33)						
Belgium	5	(0.11)					5	(0.33)						
France	3	(0.07)	2	(0.14)			1	(0.07)						
Spain	1	(0.02)					1	(0.07)						
Sweden	1	(0.02)							1	(0.07)				
**Asia**	269	(1.98)	227	(6.10)			22	(1.28)					20	(0.66)
China	92	(34.20)	85	(37.44)			7	(31.82)						
Taiwan	73	(27.14)	73	(32.16)										
Thailand	57	(21.19)	53	(23.35)			4	(18.18)						
Japan	32	(11.90)	4	(1.76)			8	(36.36)					20	(100)
Lebanon	9	(3.35)	6	(2.64)			3	(13.64)						
Laos	3	(1.12)	3	(1.32)										
Cambodia	2	(0.74)	2	(0.88)										
Viet Nam	1	(0.37)	1	(0.44)										
**Oceania**	202	(1.48)	191	(5.13)			10	(0.58)					1	(0.03)
Australia	202	(100)	191	(100)			10	(100)					1	(100)
**South America**	93	(0.68)	2	(0.05)	2	(0.06)	27	(1.57)	9	(0.66)	24	(7.00)	29	(0.95)
Chile	43	(46.24)					18	(66.67)					25	(86.21)
Brazil	27	(29.03)					9	(33.33)			18	(75)		
Ecuador	16	(17.20)			1	(50)			9	(100)	6	(25)		
Guyana	3	(3.23)											3	(10.34)
Colombia	2	(2.15)	2	(100)										
Barbados	1	(1.08)			1	(50)								
Trinidad and Tobago	1	(1.08)											1	(3.45)
